# Special Issue: Sport Psychology Interventions for Athletes’ Performance and Well-Being

**DOI:** 10.3390/ijerph20043712

**Published:** 2023-02-20

**Authors:** Selenia di Fronso, Dagmara Budnik-Przybylska

**Affiliations:** 1 Behavioral Imaging and Neural Dynamics (BIND) Center, 66100 Chieti, Italy; 2 Department of Medicine and Aging Sciences, “G. d’Annunzio” University of Chieti-Pescara, 66100 Chieti, Italy; 3 Institute of Psychology, Faculty of Social Science, Sport Psychology Division, University of Gdańsk, 80-309 Gdańsk, Poland

Scientific evidence highlights that sport psychology interventions adopted by professionals are crucial for making a difference in athlete performance [1]. Specifically, imagery, goal-setting, self-talk, and relaxation/arousal regulation are the most common interventions adopted by practitioners and athletes to enhance performance [2]. It is also true that athletes face unique physiological and psychological stressors daily. These may contribute to injuries, overtraining, burnout, and/or other physical and mental health issues. Thus, athletes need to continuously explore interventions to counteract the detrimental effect of physical and mental tension and performing at high levels [2]. Moreover, interventions that do consider athletes’ emotional and psychological well-being are important components of high performance in sport [3]. This Special Issue of the “International Journal of Environmental Research and Public Health “aims to create a constructive discussion on up-to-date scientific data in this area”. In addition to an intriguing opinion piece in which Hsu and Tseng [4] claim that the most important attribute for the making of athletes is polished sports talent, followed by psychological, environmental, and incentive factors, we summarize in the following paragraphs the articles which have been published thus far in this Special Issue entitled, “Sport Psychology Interventions for Athletes’ Performance and Well-Being”.

Athlete burnout is one of the most frequent consequences of poor mental health, as well as one of the most discussed topics among sport psychologists. Interventions to reduce or mitigate this issue are still under investigation; thus, Wilczyńska et al. [5] conducted a systematic review with a meta-analysis to examine the psychological interventions carried out to help young athletes with burnout syndrome. To this purpose, scientific electronic databases (e.g., Web of Science, PubMed, and Google Scholar) were searched, and five studies published between January and June 2022 that met the eligibility criteria (i.e., at least one treated and one control group with pre- and post-test measures; randomized controlled trials; participants that were young athletes with a maximum age of 25 years; and a mental intervention carried out with outcomes on the basis of burnout data for which an effect size could be calculated) were selected. The authors found that cognitive behavioral therapy- and mindfulness-based interventions, especially those held online, effectively reduced most dimensions of burnout. However, they concluded there should be more high-quality studies on this topic, as burnout can lead to deleterious physical and psychological problems not only for athletes, but also for their coaches. Accordingly, mental health and burnout problems require specific interventions and prevention strategies.

Regarding mindfulness-based interventions, di Fronso and colleagues recently verified the effectiveness of a mindfulness-based stress reduction (MBSR) program in sport and physical activity domains [6]. MBSR includes practices such as mindful yoga, body scanning, and sitting meditation. The authors examined the effects of a dynamic and a static strategy on psychobiosocial states (PBS), perceived stress (PS), and mindfulness levels in athletes and recreationally active (RA) people. In particular, the dynamic strategy was represented by mindful yoga while the static one was represented by a combination of body scanning and sitting meditation. In total, 34 participants (athletes = 18; RA participants = 16) were included in the dynamic intervention strategy, and the other 34 (athletes = 19; RA participants = 15) were included in the static intervention strategy. Before, after the intervention, and three weeks later, the Italian versions of the PBS scale, the PS scale, and the mindful attention awareness scale were completed by participants. The analyses of variance showed that both intervention strategies improved functional PBS, reduced PS, and enhanced mindfulness levels in both athletes and RA individuals after the intervention. On the other hand, improved functional PBS after the intervention and stable PS levels at follow-up were observed mainly in athletes. These findings underline the importance of the body to support emotional and health processes, and encourage the use of mindfulness strategies in sport to improve individuals’ well-being. It should be noted that long-term programs are recommended in RA participants as the effects of the mindfulness strategies seem to be less impactful and enduring.

Moreover, Tebourski et al. [7] conducted a study about Mindfulness For Performance (MFP). Inspired by MBSR and acceptance commitment therapy, and regardless of the disruptive sensations and thoughts induced by the performance situation, MFP is conceived to help athletes maintain effective attentional focus. It encompasses: (1) psychoeducational content and identification of the focus of attention, (2) mindfulness and acceptance training, and (3) the integration of the skills acquired in training and competition. In particular, the authors reported the effects of MFP in two studies: one about national basketball players and the other one in young table tennis players. The study with basketball players showed that mindfulness skills and free-throw accuracy during games improved more in the experimental group. In addition, the study with table tennis players highlighted that individuals who showed the highest percentage of adherence to the program highly benefited from MFP training in terms of ranking points. Despite the fact that more research with different performance indicators is necessary, both studies provided initial evidence on the promising effects of MFP on performance.

The basketball–mindfulness relationship was examined also by Wang et al. [8]. In fact, the authors adopted a quasi-experimental design to study the effect of a 7-week mindfulness intervention on the psychological coping ability and shooting performance of college-level male basketball athletes in Macau. To carry out this study, 43 male college basketball athletes were recruited. Besides regular basketball training, the intervention group (n = 23) received mindfulness training for 7 weeks. On the contrary, the control group (n = 20) received no mindfulness training. Before and immediately after the intervention, all athletes filled out the five-facet mindfulness questionnaire, the acceptance and action questionnaire, the sport competition anxiety test, and the mindfulness attention awareness scale, and also performed three shooting tests. The intervention yielded improvements in mindfulness level, acceptance level, and attention level, as well as in the three-point and free-throw shooting performance in participants who received mindfulness training. While further studies are necessary, the present research corroborates the importance of mindfulness training for psychological outcomes and shooting performance enhancement in (Macau college) basketball athletes.

Following the principles of self-regulation, Ruiz et al. [9] used an action and emotion regulation-based program in ice hockey players. The authors’ hypothesized that the intervention would help players self-regulate their core action components and PBS states. Ice hockey junior players were recruited from two teams competing at the highest level of the Finnish league, and were then assigned to a self-regulation and a control group. Specifically, the self-regulation program adopted by the authors in the experimental group targeted the recreation of the optimal execution of core action elements and functional feeling states. Additionally, it included elements such as imagery and slow-paced breathing. On the other hand, participants in the control group had to follow a breathing pattern similar to spontaneous breathing. A significant increase in vagal tone was observed for the participants in the intervention group. However, the results did not yield significant differences between the self-regulation and control groups in the accuracy and control ratings of the selected action elements, or in the intensities of the feeling states associated with actual performances. The repeated assessments of the aspects related to the action and experiences of the participants likely enhanced the awareness in all participants. Participants in the control group probably used their own self-regulation strategies. The authors also speculated that individual profiles based on action components and psychobiosocial states induced self-reflection, which increased players’ awareness of functional and dysfunctional states and stimulated the identification/initiation of self-regulation strategies. The findings still encourage the combination of strategies targeting the regulation of core action elements and feeling states.

Strategies to enhance self-confidence are generally considered to be common sport psychology interventions for athletes’ well-being and performance, yet evidence of the relationship between self-confidence and athletic performance is equivocal. As a consequence, Lochbaum and colleagues [10] conducted a systematic review with a meta-analysis on this topic. Moreover, they examined potential risk-of-bias indicators, as well as the moderation effects of study quality, sport characteristics, the timing of confidence measurements, and individual differences among participants. To this purpose, and in addition to some searching by hand, the authors reviewed two past meta-analyses and systematically searched APA PsycArticles, ERIC, Psychology and Behavioral Sciences Collection, PsychINFO, and SPORTDiscus within the EBSCOhost platform. Specifically, studies that met the eligibility criteria showed that the self-confidence/performance relationship is small in magnitude, nearly free of bias, and moderated by sport type, performance objectivity, and athlete gender and, consequently, it should be revisited. Indeed, although it might be true that without confidence, athletes cannot win, it might be more precise that without more confidence than the other team or competitors at a critical moment, it is easier not to win.

Li et al. [11] likely based their contribution to this Special Issue on the idea that interventions to improve sport performance boost athletes’ mental health. Indeed, the authors systematically reviewed the literature about the effects of attentional focus on sprint performance, adopting a meta-analytic technique. Precisely, they reviewed existing findings on the impact of external focus (EF) in comparison to internal focus (IF). They purposefully screened databases such as APA PsycINFO, PubMed, Scopus, SPORTDiscus, and Web of Science. Their findings revealed that the EF condition can be considered by far better than the IF condition in sprint performance. However, the subgroup analysis should be viewed with caution and although no significant difference was found between the considered subgroups, the benefits associated with the EF strategy seemed to be significant in low-skill sprinters, but not significant in high-skill sprinters. While corroborating the EF effects, Li and colleagues concluded that making adjustments in verbal instructions can lead to significant behavioral effects of great importance in competitive sports and that the reported gain in sprint performance due to attentional focus has practical implications for coaches and athletes. In particular, given that a 1% improvement can increase the chances of success and foster medal positions in international competitions, athletes and coaches should design EF strategies in accordance with their skill development to improve sprinting. 

Overall, this Special Issue offers a well-arranged view on sport psychology interventions for athletes’ performance and well-being. To date, the emerging trends shed light on: programs to reduce athletes’ burnout (e.g., behavioral therapy and mindfulness-based interventions); static and dynamic mindfulness-based strategies to reduce stress and improve emotions in athletes of different levels, and specific mindfulness interventions for sport performance (i.e., in basketball and tennis),and psychological coping abilities; the effectiveness of a self-regulation program that includes action and emotion regulation strategies for ice hockey athletes; an updated overview of the relationship between self-confidence and athletic performance, the utmost importance of EF for sprinters. Beyond systematic reviews with meta-analyses and an opinion paper, this Special Issue mainly consists of original research articles. Altogether, these studies, due to their uniqueness, may offer ideas for athletes, coaches, and researchers working in this field to use innovative interventions/research paradigms with the purpose of further strengthening athletic achievements and health. 
